# The relationships between social media exposure, food craving, cognitive impulsivity and cognitive restraint

**DOI:** 10.1186/s40337-022-00698-4

**Published:** 2022-11-25

**Authors:** Lisa Filippone, Rebecca Shankland, Quentin Hallez

**Affiliations:** 1 Laboratoire Développement, Individu, Processus, Handicap, Éducation (DIPHE), Institut de Psychologie, Université Lumière Lyon 2, 5 avenue Pierre Mendès-France, Bron, France; 2 Institut Universitaire de France, Paris, France

**Keywords:** Social media, Cognitive impulsivity, Food craving, Cognitive restraint

## Abstract

**Background:**

Young adults are increasingly exposed to social media and their image/video-based activities. They use these platforms to share images, videos and advice in different fields like food and nutrition with: recipe ideas, nutritional opinions or specific diets. Along with the rise of digital technologies, the prevalence of eating disorders in young adults continues to grow. The present study analyzes the psychological and eating processes through which exposure to social media may lead to the development of food craving and problematic eating behaviors.

**Methods:**

A total of 103 young adult men (n = 15) and women (n = 88) answered questionnaires measuring their impulsivity (Barratt Impulsiveness Scale), eating habits (Three-Factor Eating Questionnaire), food craving (Food Cravings Questionnaire-Trait-reduced), and time exposure to social media.

**Results:**

The results showed two significant serial mediations. We found a correlational link between time exposure to social media and food craving scores. This positive relation is indirectly mediated by cognitive impulsivity. We also found a positive correlation between cognitive impulsivity and food craving scores that was mediated by cognitive restraint.

**Conclusion:**

A better understanding of the existing links between social media, food craving and eating behaviors such as cognitive restraint could help researchers and clinicians to better guide young adults in their use and appropriation of social media food contents.

## Plain English summary

Young adults are increasingly using social media to share images, videos and opinions on a wide range of areas including food. Young people discuss recipes tips, nutritional opinions or specific diet advice. Online platforms expose young people to a set of contradictory data, often generalist, and non-individualized advice that can create the feeling of being overwhelmed by information. This study aimed to understand the potential impact of this increasingly frequent exposure to social media on eating behaviors of young adults. The results showed that longer exposure times to social media were indirectly linked to an irrepressible urge to eat via increased impulsivity. High impulsivity scores were also indirectly related to food craving via food restriction. A better understanding of the links between social media, food craving and eating behaviors could help researchers and clinicians to better guide young adults in their use and appropriation of social media food contents. By knowing more precisely the processes at work behind exposure to social media, it can become possible to undertake awareness actions in order to prevent the development of subsequent eating issues.

## Background

In 2019, 94% of young people aged 16 to 29 in the 27-member states of the European Union, made daily use of the internet [27], with an average of 1 h 15 min spent per day on social media [10]. With a lifespan estimated at 73.4 years in 2019 by the WHO [106], individuals will spend around 6 years and 8 months of their lifetime on social media [10]. Although traditional media (e.g., television, magazines) are still watched, social networks (e.g., Facebook, Instagram, Twitter) are taking an increasing share of this cut of daily exposure times [82], to such an extent that these social platforms are considered by young adults aged 18 to 25 as an integral part of their lives [19].

Among other things, young adults perceive social media as a platform to share information about food [110]. By communicating and sharing about the food they eat, they may mutually influence their eating habits. For example, the perception that peers often consume sugary drinks and pastries has been shown to predict the consumption of these products among young adults [74]. Furthermore, social interactions can influence food choices in terms of products and portions chosen by young adults [57]. Social media also largely convey cultural ideals regarding attractiveness and thinness trough images and videos which can lead to body shame and appearance anxiety [9]. In young adults under the age of 25, exposure to this type of content is associated with body dissatisfaction that can lead to compulsive practice of physical exercise related with excessive dietary control [14]. These body issues could motivate restrained eating in an effort to lose body fat to appear further in line with the thin female ideal [20]. Indeed, a recent systematic review by Rounsefell et al. [76] showed that social media engagement negatively impacted body image and food choices in healthy young adults. Thus, a link has been reported between social media and food behaviors. The model we propose in this study attempts to explain how exposure to social media can be linked to food craving. Food craving is defined by a strong and irresistible desire to consume a specific type of food 15], and a psychological and physiological motivational state that leads to the consumption or ingestion of the desired substance [59]. We expect that exposure to social media will be associated with participants’ impulsivity and in particular the cognitive dimension of impulsivity, defined as the inability to inhibit behavioral impulses and thoughts [6]. This cognitive impulsivity could then be linked to dysfunctional eating behaviors such as cognitive restraint. Cognitive restraint is a set of cognitively guided behaviors implemented to decrease food intake [28]. Ultimately, the maintenance and repetition of these behaviors could be associated with food craving and eating disorders (EDs) in young adults.

### Social media and food craving

Social media can project normative ideas about what constitutes a healthy meal and simultaneously infer which foods should be consumed in a limited way [44]. There is thus a possible impact of “clean eating” (e.g., eating local, unprocessed, organic, plant-based and homemade foods; Dennett [22]) proponents who actively promote this content via social media like Instagram [1]. “Clean eating” contents, in their extreme forms may have negative consequences such as the development of eating disorder risk factors like cognitive restraint. The restraint model of binge eating [70] indicates that the risk of binge eating increases when individuals experience moments of temporary inattention in the cognitive control of their eating. Binge eating is defined by eating a large amount of food in a limited period of time, with a co-occurring feeling of loss of control [3]. Mechanisms that explain these moments of inattention and decreased focus in cognitive control of eating appear to include the “abstinence violation” effect that occurs when someone violates a dietary rule [54])or because of a depletion of limited cognitive resources for self-regulation [63]. The goal conflict model Stroebe et al. [86] suggests that the eating behavior of restricted eaters is dominated by a conflict between their weight control goals and the pleasure of eating: as soon as the goal of enjoying palatable food is activated in restricted eaters then the conflicting goal of controlling weight is inhibited. This mechanism underlies the self-regulatory failure of restricted eaters in environments where attractive food is readily available. Massey and Hill [56] observe that, in comparison with individuals who do not diet, dieters experience more intense food craving which is difficult to resist, especially for foods that they greatly restrict themselves to eat.

The potential consequences of social media might be increased by the time of exposure to screens. Among students, problematic internet use (i.e., uncontrolled use of Internet that affects the user’s behavior, with a concurrent loss of self-control and an increase in impulsivity) is linked to the development of eating disorders [40]. Recently, Wilksch et al. [103] showed that greater daily time spent using Instagram in adolescent girls was associated with significantly higher disordered eating behaviors. In addition, nocturnal exposure to the blue light from screens leads to a change in glucose metabolism, which may be responsible for the development of obesity in adults [31]. Social media also represent a new marketing opportunity for food and beverage companies, that widely use media via influencer advertising (i.e., personalities collaborate with brands to endorse and promote products to their followers; Veirman et al. [99]). Associations exist between influencer marketing of food and beverage products and various viewer behaviors, including brand recognition, desire to like and share posts and consumption of the products [80]. The protective advertising disclosure policies set up by governments may even increase the magnitude of food intake response [17]. Recently researchers showed that among 621 Twitch users, 72% recalled observing at least one food or beverage advertisement on Twitch. After observing advertised products, 14% reported craving for the product, and 8% reported purchasing one [71]. A significant positive correlation was found between the level obesity rate and the mean percentage of followers of sugary drink or fast-food brands on Instagram and Twitter [36]. Engagement with unhealthy food brands on social media is common among adolescents [30]. Exposure to this marketing increases preferences and consumption of these products [42]. Thus, exposure to fatty and sugary products is concurrent with exposure to the rigid food rules promoted by “clean eating” contents. The combination of these two different concepts can create an overwhelming digital environment regarding food choices and rules. Finally, it is important to note that social media have been related not only to food craving, but also impulsive behaviors, which may intersect and contribute to eating behavior difficulties.

### Social media and impulsivity

Research has suggested that impulsivity is a major risk factor for the development and maintenance of social media use disorders [75, 91]. Repeated use of social media via smartphones and heightened reward expectations can lead to habitual, everyday use and impulsive responses to social media-related material [35, 95, 96]. More specifically, this repeated use can lead to the emergence of addictive behaviors with loss of control over consumption, despite the negative consequences [95]. Although there is no official classification of social media use disorder yet, researchers point out that these addictive-type behaviors may be comparable to other behavioral addictions [50, 61, 101]. Worsley et al. [107] consider such behaviors as problematic use of social media, while Bilgin and Tas [8] suggest the existence of a social media addiction. This relationship between social media and impulsivity is also found at the neurological level. Researchers found an association between neuro-anatomical correlates of addiction to social networks and a reduction in the gray matter of an amygdala’s region involved in the generation of impulsive behaviors, as in other types of addiction [39].

Social media, with their frantic pace, may increase an impulsive cognitive style to be able to repeatedly and constantly reorient attention on the newest stimulus presented Nikkelen et al. [66]. It could therefore suggest that it is more precisely the cognitive dimension of impulsivity (i.e., inability to inhibit behavioral impulses and thoughts) that would be affected during exposure to social media. Indeed, the behavioral addiction theories suggest that a set of cognitive-emotional deficits in terms of reward processing and inhibition, automaticity and lack of prefrontal oversight, and cognitive dysregulation can explain addictive behaviors [7]. These cognitive deficits are rooted in a dual system: hyperactivity of an impulsive system which enables quick responses towards immediately gratifying options and hypoactivity of the cognitive control processes of a reflective system that governs decision making, executive functions and inhibitory control [92]. This literature confirmed our choice of a measure targeting the cognitive dimension of impulsivity.

### Impulsivity and food craving

Impulsivity is also considered to be an etiological and/or probable maintenance factor for binge eating behaviors [34, 108]. The tendency to act impulsively when under distress, positively predicted symptom count of food addiction in undergraduate students 69]. Food addiction can be characterized by symptoms such as loss of control over consumption, continued use despite negative consequences, and an inability to cut down despite the desire to do so [33]. At the neurological level, impulsive food choices are associated with activation in brain areas involved in reward processing [46, 94]. Thus, individuals with binge-eating disorders have limited resources to inhibit their reaction to a food stimulus, and this phenomenon is further marked in individuals with high trait-impulsivity [100]. Interventions related to food impulsivity seem to be promising in increasing the control of inhibition and reducing the frequency of binge-eating behaviors and food craving [41].

### Impulsivity and cognitive restraint

Impulsivity also seem to be linked to food intake, and especially for restrained eaters. Guerrieri et al. [37] found that the caloric intake of participants was significantly higher in an “impulsivity induction” condition compared to an “inhibition induction” condition. This effect was even stronger for highly restrained participants. Thus, an impulsivity induction in restrained individuals lead them to overeat. According to Stice [85] and his dual pathway model of bulimia, the increase in restrictive behaviors is linked to an increase in uninhibited eating behaviors due to physiological hunger and strong food craving. Thus, women with high levels of negative affect, high food restriction and high cognitive impulsivity have higher levels of binge eating [55]. In a recent study, researchers showed that, in individuals with eating disorders in the binge eating frequency spectrum, increased food restriction is a mediator of the relationship between high levels of impulsivity and frequent binge eating episodes [60].

With the rise of digital technology and the 8% increase in the prevalence of eating disorders since 2013 [32], the question of the role of social media in our eating behaviors has become a public health issue. A better understanding of the links between social media, food craving and eating behaviors such as cognitive restraint will help researchers and clinicians to better guide young adults in their use and appropriation of social media food contents. By knowing more precisely the processes at work behind exposure to social media, it can become possible to undertake awareness actions in order to prevent the appearance of subsequent eating issues. The period of transition to adulthood is decisive in the construction of young people, who learn to develop sustainable health behaviors at this age, particularly in the area of ​​eating habits [64]. That is why, nowadays, the increased use of social media and their possible harmful consequences on eating habits should be considered seriously. Indeed, we found no research that studied social media exposure, cognitive impulsivity, eating behaviors and food craving in combination. Note that we will not assess the presence of problematic use of social media (PUI) but only the time of exposure to social media, which are to distinct concepts. This research focuses on a student population, a priori healthy, so that an addiction measure could have not been appropriate. The assessment of the time spent in front of social media seemed to be a good alternative in order to take into account all the distinctive feature of the entire population. The aim of the present study is to test two models of mediation. First, we expect that (hypothesis 1, mediation 1) greater exposure to social media would lead to increasing food craving, and that the link would be mediated by cognitive impulsivity. Next, we expect that (hypothesis 2, mediation 2) the link between cognitive impulsivity and food craving would be mediated by cognitive restraint.

## Methods

### Population

One hundred and fifteen individuals took part in this cross-sectional survey. The exclusion criteria were a lack of consent and not having fully completed the survey. Five participants were removed from further analyses as they were outliers in their use of the social media Instagram (either they did not use it at all, or too little or a lot compared to the average). We detected the outliers using the box and whiskers option of JASP.

The final sample consisted of 103 participants; 88 of whom were female (86%). The participants ranged in age from 18 to 61 years (*M* = 29.32, *SD* = 13.51). They were recruited to take part in a study on “time, eating behaviors and social media” as part of a larger scale study. They were enrolled via an invitation communicated in the psychology courses of their university and published via Social Media (Facebook). Participants gave their consent via an electronic form presented at the beginning of the online experiment. The experiment was carried out in accordance with The Code of Ethics of the World Medical Association (Declaration of Helsinki) for experiments involving humans. The ethics committee group of the University of Nantes approved the ethic of this study.

### Material and procedure

The survey was conducted using Qualtrics® electronic survey software, which recorded the participants’ responses and randomly delivered the questionnaires.

#### Demographics

Participants were asked their gender and year of birth.

#### Social media time exposure

Five items were designed by the authors for the purpose of the study. Participants were first asked if they use social media (yes or no). They were then asked to rate their use of social media in general (“Estimate the average time you spend on social media in general”) and then more specifically of Instagram (“Estimate the average time you spend on Instagram”). For both of these two statements they had to answer on two 360-point self-evaluation scale ranging from 0 to 360 min (one scale for the time spend during the week and the other during the week-end). We then averaged time spent on weekdays and weekends to get a measure of average time spent on social media (SocialMediaTime) in general and a measure of average time spent on Instagram (InstaTime).

#### Impulsivity

Impulsivity was evaluated using the French version [77] of Spinella’s [83] 15-item Barratt Impulsiveness Scale (BIS-15). Participants had to answer on a 9-point Likert Scale (from 0 “not like me at all” to 9 “exactly like me”) to 15 sentences evaluating their ways of acting and thinking in various situations (e.g., “I am a thoughtful person”, “I buy things on a whim”). This instrument gives a global impulsivity score, and 3 other scores based on 3 subscales: lack of planification (lack of planification of the future), cognitive impulsivity (tendency to take quick decisions), and motor impulsivity (tendency to act without thinking). Based on previous studies, the reliability of the scales is relatively satisfactory with Cronbach’s alphas between 0.81 and 0.68. As explained in the introduction, we decided to focus our analyzes only on the cognitive dimension of impulsivity.

#### Food craving

Food craving was measured using the 15-item-French version of the Food Cravings Questionnaire-Trait-reduced (FCQ-Tr, Meule et al. [58], French version: Brunault et al. [11]). Participants rated the frequency with which they engage in food craving behaviors using a 6-point scale ranging from “Never = 1” to “Always = 6”. Based on previous studies, this scale has a one-factor structure (that explained 58% of the total variance) with high factor loadings for all items (> 0.50) and a high internal consistency (*α* = 0.95).

#### Eating behaviors (cognitive restraint, uncontrolled eating and emotional eating)

The eating behaviors were described using a French Translation of the Three-Factor Eating Questionnaire TFEQ-R21 [12]. The instrument is a shortened and revised version of the original 51-item TFEQ [87]. Responses to 20 items are rated from 1 to 4 and item scores are summated into 3 subscales, namely: cognitive restraint (high score on this subscale highlights a conscious restriction of food intake in order to control body weight or to promote weight loss), uncontrolled eating (high score on this subscale indicates a tendency to eat more than usual due to a loss of control over intake accompanied by subjective feelings of hunger), and emotional eating (high score on this subscale points out an inability to resist emotional cues). Item 21 comes with a 1 to 8 scale from “no restriction” to “very high restriction” that is then transformed to give a 1 to 4 score. All the raw scale scores are transformed to a 0–100 scale [((raw score _ lowest possible raw score)/possible raw score range) × 100] and the commonly used “half-scale” method is used to compensate for missing data on some items. Higher scores in the respective subscales are indicative of greater cognitive restraint, uncontrolled, or emotional eating. Based on previous studies, the reliability of the subscales is satisfactory with Cronbach’s alphas between 0.92 and 0.68.

## Statistical analyses

**Fig. 1:**
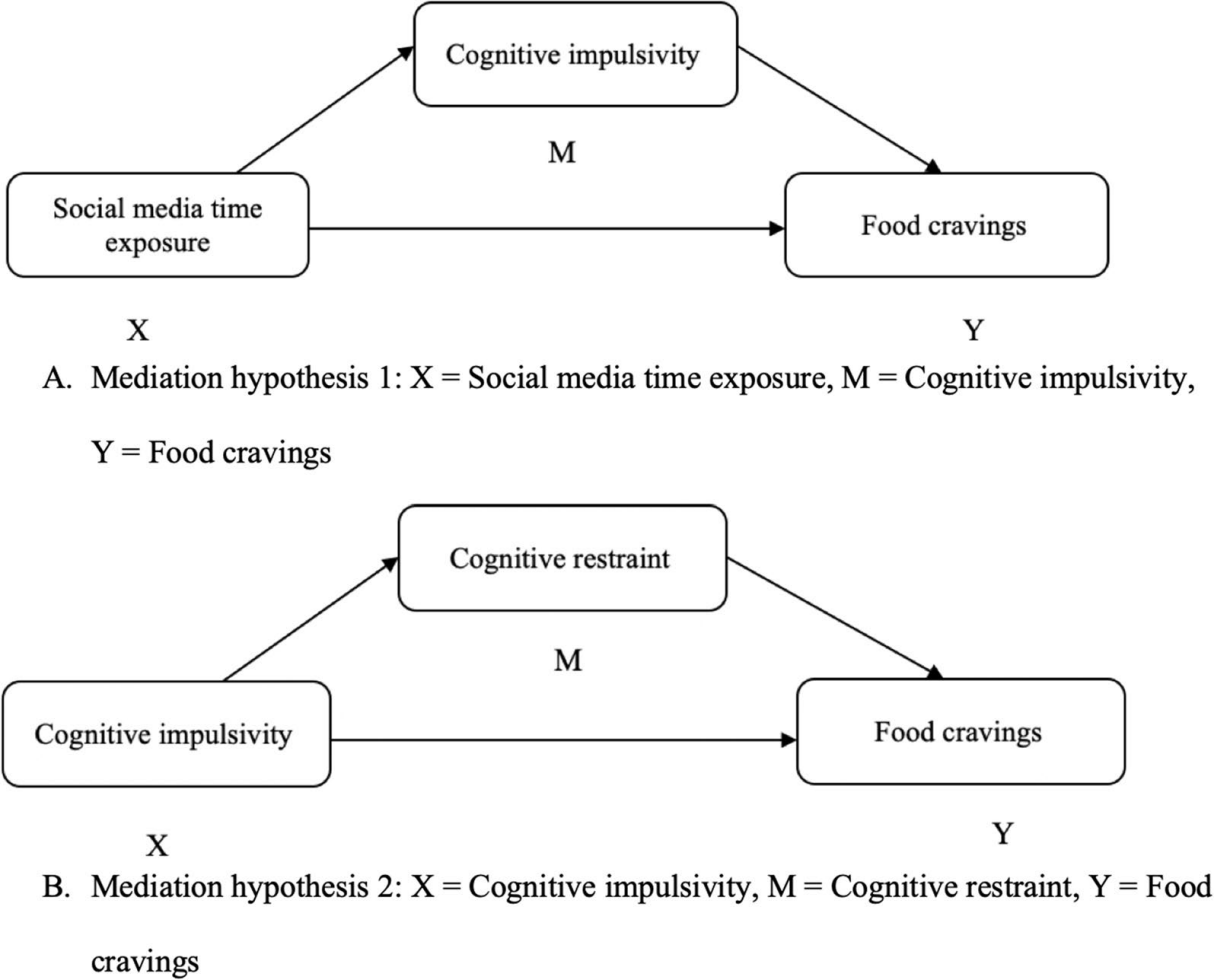
Two mediation hypotheses

Data were analysed using JASP 0.15 (JASP Team, 2021) and SPSS v. 26 (IBM, 2019). First, descriptive statistics (means and standard deviations were computed. All the skewness and kurtosis values were included between − 2 and 2, which is considered acceptable in order to prove normal univariate distribution [48, 97]. Pearson’s correlations were conducted for each variable of interest to describe the data and the relationships between the factors. Regressions analyses of the temporal variables (social media time exposure) on the eating (emotional and uncontrolled eating, cognitive restraint and food craving) variables and cognitive impulsivity were conducted. Regression analyses were also launched between the different eating variables and cognitive impulsivity. Finally, we launched a set of 2 mediation analyses to test our 2 mediations (Fig. 1). All mediation models were tested using Process SPSS macro model 4, and a bootstraps sample of 5000 [72].

## Results

### Preliminary analyses

Table 1 shows the descriptive statistics for the social media time exposure, the cognitive impulsivity, the eating variables and age.

**Table 1:** Mean, standard deviations, skewness and kurtosis associated with social media time exposure, cognitive impulsivity subscale of the Barratt Impulsiveness Scale (BIS), uncontrolled eating, emotional eating, cognitive restraint, food craving, and age

	Mean	SD	Skewness	Kurtosis
Social media time exposure	136.07	84.73	0.63	− 0.33
BIS cognitive impulsivity	20.71	8.41	0.27	− 0.29
Uncontrolled eating	40.24	24.35	0.26	− 0.89
Emotional eating	128.91	64.55	0.14	− 1.14
Cognitive restraint	34.57	23.96	0.23	− 0.89
Food craving (FCQTr)	44.48	15.01	0.36	0.01
Age	29.32	13.51	1.06	− 0.33

### Correlation analyses

Table 2 presents the Bravais-Pearson’s correlations between the social media time exposure, eating variables and cognitive impulsivity.

**Table 2:** Bravais Pearson's correlation analyses of the cognitive impulsivity subscale of the Barratt Impulsiveness Scale (BIS), uncontrolled eating, emotional eating, cognitive restraint, food cravings (FCQTr) and social media time exposure

		BIS cognitive impulsivity	Uncontrolled eating	Emotional eating	Cognitive restraint	Food cravings (FCQTr)	Social media time exposure
BIS cognitive impulsivity	Pearson's r	–					
	*p* value	–					
Uncontrolled eating	Pearson's r	0.33***	–				
	*p* value	<. 001	–				
Emotional eating	Pearson's r	0.26*	0.68***	–			
	*p* value	0.01	< .001	–			
Cognitive restraint	Pearson's r	0.12	0.27*	0.26*	–		
	*p* value	0.21	0.01	0.01	–		
Food cravings (FCQTr)	Pearson's r	0.39***	0.77***	0.70***	0.32**	–	
	*p* value	< .001	< .001	< .0.01	0.001	–	
Social media time exposure	Pearson's r	0.24	0.29**	0.10	0.06	0.26*	–
	*p* value	0.02	0.003	0.32	0.58	0.01	–

*p < .05, **p < .01, ***p < .001

We found that cognitive impulsivity was positively linked to the social media time exposure (*p* = 0.02). Cognitive impulsivity was positively related to uncontrolled eating (*r* = 0.33, *p* < 0.001), emotional eating (*r* = 0.26, *p* = 0.01), and food craving (*r* = 0.39, *p* < 0.001). The food craving scores were positively related to uncontrolled eating (*r* = 0.77, *p* < 0.001), emotional eating (*r* = 0.70, *p* < 0.001), and cognitive restraint (*r* = 0.32, *p* = 0.001).

### Mediation analyses

#### Mediation 1) social media time exposure, food craving and cognitive impulsivity

First, we found a trend (non-significant) direct relation between social media time exposure and food craving (*t* = 1.82, *β* = 0.03, *SE* = 0.17, 95% CI [− 0.03, 0.07], *p* = 0.07). However, social media time exposure was associated with the cognitive impulsivity scores (*t* = 2.40, *β* = 0.02, *SE* = 0.01, 95% CI [0.004, 0.04], *p* = 0.02). In addition, cognitive impulsivity scores were also linked to food craving scores (*t* = 3.65, *β* = 0.62, *SE* = 0.17, 95% CI [0.28, 0.97], *p* < 0.001). The mediation model revealed that the link between social media time exposure and food craving decrease when the mediator (cognitive impulsivity) was taken into account. Preacher and Hayes’s bootstrap method confirmed the significance of the indirect effect of cognitive impulsivity (*B* = 0.01, *SE* = 0.01,* 95% CI* [0.004, 0.04]) on the relation between social media time exposure and food craving.

### Mediation 2) cognitive impulsivity, food craving and cognitive restraint

We found a significant direct relation between the cognitive impulsivity and the food craving scores (*t* = 3.17, *β* = 0.49, *SE* = 0.15, 95% CI [0.18, 0.79], *p* = 0.002). In addition, cognitive impulsivity was also associated with the cognitive restraint scores (*t* = 2.31, *β* = 0.59, *SE* = 0.26, 95% CI [0.08, 1.11], *p* = 0.02), the latter being significantly linked with the food craving scores (*t* = 2.70, *β* = 0.16, *SE* = 0.06, 95% CI [0.04, 0.27], *p* = 0.01). The mediation model analysis showed that the significant relation between cognitive impulsivity and food craving decrease when the mediator (cognitive restraint) was taken into account. With the use of the Preacher and Hayes’s bootstrap method we confirmed the significance of the indirect effect of cognitive restraint (*B* = 0.09, *SE* = 0.06, *95% CI* [0.01, 0.25]) on the relation between cognitive impulsivity and food craving.

**Fig. 2:**
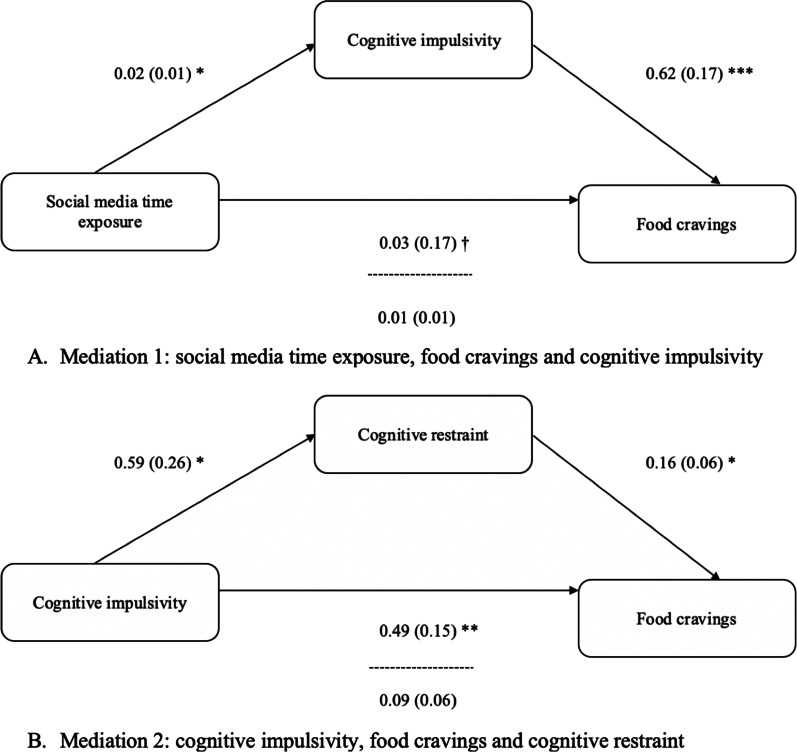
The mediation models (**A**,** B**). *p < 0.05, **p < 0.01, ***p < 0.001

Figure 2 shows both mediation models.

## Discussion

The aim of the present study was to analyze the psychological and eating processes through which exposure to social media can lead to the development of food craving and cognitive restraint. To our knowledge, our study was the first to investigate the links between social media exposure time, cognitive impulsivity and eating behaviors. Thus, in line with hypothesis 1, a higher exposure to social media was related to cognitive impulsivity. These results corroborate those of neuro-anatomic studies that found changes in the cerebral structures involved in cognitive impulsivity in individuals suffering from internet addiction [21, 53, 90]. In our study, higher levels of individual cognitive impulsivity as well as uncontrolled eating were associated with food craving. These results are not surprising since impulsivity is known as an etiological and/or probable maintenance factor for binge eating behaviors [34, 108]. More precisely, our study showed that it is the cognitive aspect of impulsivity that was linked to disordered eating patterns. In fact, problematic eating behaviors (e.g., binge eating) are associated with alterations regarding conflict processing and inhibitory control deficits [51, 89]. Studies that investigated the neurobiological basis of binge eating reported an enhanced attentional bias towards food stimuli, alterations in the reward system, and impairments in cognitive functions (e.g., poor inhibitory control skills towards food; Kessler et al. [47], Smith et al. [81]). Thus, Wolz et al. [104] showed that training inhibitory control through behavioral inhibition might be effective in reducing subjective craving and food intake. Our first mediation model showed no significant direct link between exposure to social media and food craving. When analysing the indirect effect, the mediation model revealed that the time individuals spent on social media was linked to food craving through higher levels of cognitive impulsivity. Our findings contrast with the meta-analysis by Zhang et al. ([109]) which showed that excessive use of Social Networking Sites (SNSs) was associated with an increased risk of disordered eating behaviors, and that university students showed a larger correlation between SNS use and disordered eating behaviors than other samples. The fact that in our sample no direct relation was found may be explained by the fact that the participants may have had less excessive use of social media than in other samples of the previously published studies. Furthermore, it could be due to the fact that we did not control for the exposure to food cues in these social media. Therefore, in our study, it was more specifically cognitive impulsivity that was related to food craving. Indeed, the second mediation model (hypothesis 2) showed a significant relation between cognitive impulsivity and food craving scores. In addition, cognitive impulsivity was also associated with cognitive restraint scores, and the cognitive restraint scores were significantly linked with the food craving scores. Moreover, the significant link between cognitive impulsivity and food craving decreased when the cognitive restraint mediator was taken into account. In other words, we found that the higher the cognitive impulsivity levels were, the more they showed food craving, and this relation was explained by higher levels of cognitive restraint. This can be explained by the fact that, in our study, the restriction can be seen as an adaptive behavior since the participants are part of a non-eating disordered sample, who should be less likely to engage in dieting behaviors. Guerrieri et al. [37] found that restrained and unrestrained nondieters had significantly higher caloric intake when impulsivity was induced compared to inhibition. Conversely, they showed that current dieters reduced their caloric intake following the impulsivity induction. For these researchers, restriction and dieting are two different constructs that do not affect the regulation of food intake in the same way. Thus, according to them, it would be intentional dieting that would lead to a significant decrease in caloric intake and not the simple fact of occasionally restricting oneself. Individuals with an eating disorder characterized by binge eating and who demonstrate high impulsivity may make rash decisions to adhere to strict dietary rules to compensate for the calories of a previous binge eating episodes [13]. Individuals who do not diet will then probably not fall into the impulsive spiral of an ever more intense and controlling eating restriction that would lead them to a more disinhibited diet [18].

The strength of this study is that we managed to present indications of a cross-sectional relationship between social media time exposure and food craving through an increase in cognitive impulsivity levels. Interestingly, exposure to social media did not have to present addictive features for significant results to be observed. Furthermore, we found that the link between cognitive impulsivity and food craving can be lessened by restrictive behaviors. This second mediational finding is indirectly linked to the first one as we succeeded to found a correlational relationship between social media exposure time and cognitive impulsivity.

Nevertheless, we have to note that there are some limitations to the present work. First of all, we didn’t control the presence of eating disorders or other physical or mental affections in our participant’ sample. Despite the fact that we did not question the presence of eating disorders, our attempts to hide the objectives of the study would have benefited from being more advanced in order not to induce a social desirability bias in participants’ responses. We didn’t know either if the participants were possibly current or former dieters (i.e., we didn’t know if during the period of the study participants considered that they were dieting). Furthermore, the sample was small and likely to be underpowered to find the often small effect sizes usually found in the social media literature linking social media use and eating disorders. Additionally, in the future, it would be pertinent to measure more specifically the impact of different social networks (i.e., Facebook, Instagram, Snapchat, TikTok), and to conduct research studies focused solely on exposure to social media food content. The use of a validated measure of time spent on social media could also increase the accuracy and validity of the results. In the present study, we worked on the subjective time that participants felt they spent on social media. In the future, it would be interesting to study the influence of objective social media use on cognitive impulsivity and eating behaviors. A comparison of objective and subjective social networking time could also be considered. Finally, we obtained only partial mediations, but at the same time we were able to highlight three variables (social media time exposure, cognitive impulsivity, cognitive restraint) that could, together, play a significant role on food craving. Thus, this study allowed to find several explanatory sources of food craving modulations. The above-mentioned relationships give us no information about causal relations. Thus, all the links presented can be imagined as inverted. In other words, social media consumption could generate an effect on impulsivity but it could also be cognitive impulsivity that would lead individuals to prefer social media to exchange. Similarly, it may be impulsivity that generates greater cognitive restriction, but it may also be cognitive restriction that increases cognitive impulsivity. Thus, individuals who present more food craving and therefore might have higher cognitive impulsivity levels, may choose to spend more time on social media. In fact, social media content is extremely diverse and food content is only a tiny fraction of what can be visited and preferred by young people. We can therefore imagine that in individuals without any food issues, a limited exposure to food content will not be likely to trigger the onset of disordered eating behaviors. On the order hand, a more important exposition to food content in individuals already presenting some food issues could eventually be likely to maintain or accentuate troubled eating behaviors. Examination of reciprocal relationships between the variables would require a longitudinal study with measures taken over several time points to identify the temporal sequence of relationships. In the future, several research studies should be conducted to analyse the different links between those variables and to understand what influence they respectively have on food craving and behaviors. Furthermore, variables like body mass index should also be controlled.

## Conclusion

The results of the present study showed a correlational relation between social media exposure and food craving via the increase in cognitive impulsivity in a young adult sample. Restrictive behaviors diminished the strengths of the link between cognitive impulsivity and food craving. These findings suggest that further research may be useful in this field in order to identify further potential risk factors which could explain the relationship between social media exposure and eating disorders. It may also be useful to test the potential benefits of the implementation of interventions to prevent eating disorders in young adults at two levels: on the one hand on the reduction in cognitive impulsivity that can be generated by overexposure to social media and, on the other hand, to support the development of non-restrictive eating strategies. Thus, by acting both on better management of cognitive impulsivity and excitement that can be generated by social media, and by proposing alternatives to prevent the development of problematic eating behaviours, it could be possible to limit the development of food craving in young people who are increasingly exposed to the media and prone to the development of eating disorders.

## Data Availability

Data can be reachable by sending an email to the corresponding author. If needed, the results and database can be uploaded before the second review on Open Science Framework.

## Abbreviations

BIS: Barratt Impulsiveness Scale
TFEQ: Three-Factor Eating Questionnaire
FCQTR: Food Cravings Questionnaire-Trait-reduced
WHO: World Health Organization
PUI: Problematic Use of Internet
ED: Eating disorder
SD: Standard deviation
